# Innovative Nanoparticles Enhance *N*-Palmitoylethanolamide Intraocular Delivery

**DOI:** 10.3389/fphar.2018.00285

**Published:** 2018-03-28

**Authors:** Carmelo Puglia, Paolo Blasi, Carmine Ostacolo, Eduardo Sommella, Claudio Bucolo, Chiara B. M. Platania, Giovanni L. Romano, Federica Geraci, Filippo Drago, Debora Santonocito, Barbara Albertini, Pietro Campiglia, Giovanni Puglisi, Rosario Pignatello

**Affiliations:** ^1^Department of Drug Sciences, University of Catania, Catania, Italy; ^2^NANO-i – Research Centre on Ocular Nanotechnology, University of Catania, Catania, Italy; ^3^School of Pharmacy, University of Camerino, Camerino, Italy; ^4^Department of Pharmacy, University of Naples Federico II, Naples, Italy; ^5^Department of Pharmacy, University of Salerno, Fisciano, Italy; ^6^Department of Biomedical and Biotechnological Sciences, University of Catania, Catania, Italy; ^7^Center for Research in Ocular Pharmacology, University of Catania, Catania, Italy; ^8^Bascom Palmer Eye Institute, University of Miami Health System, Miami, FL, United States; ^9^Department of Pharmaceutical Sciences, University of Perugia, Perugia, Italy

**Keywords:** palmitoylethanolamide, nanostructured lipid carriers, ocular drug delivery, retinal diseases, diabetic retinopathy

## Abstract

Nanostructured lipid carriers (NLCs) loaded with palmitoylethanolamide (PEA) were formulated with the aim to enhance ocular bioavailability of PEA, particularly to the back of the eye. Technological characterization (e.g., size, charge) of NLC loaded with PEA formulation (PEA-NLC) was performed, and NLC morphology was characterized by electron microscopy. Ocular pharmacokinetic study, after topical administration of the formulation, was carried out in rabbit eye. Ultra-high performance liquid chromatography tandem mass spectrometry analysis was carried out to detect PEA levels in ocular tissues. Finally, the ocular tolerability of PEA-NLC formulation was assessed in rabbit eye. The novel formulation significantly increased PEA levels in ocular tissues compared to PEA suspension. Vitreous and retinal levels of PEA were significantly higher in the group treated with PEA-NLC formulation versus PEA suspension (PEA-NLC C_max_ 5919 ± 541 pmol/g and 315 ± 70 pmol/g in vitreous and retina, respectively). The PEA-NLC formulation was characterized by high stability and robust ocular bioavailability. Therefore, this innovative formulation may be useful in clinical practice to manage retinal diseases.

## Introduction

**FIGURE 1:**
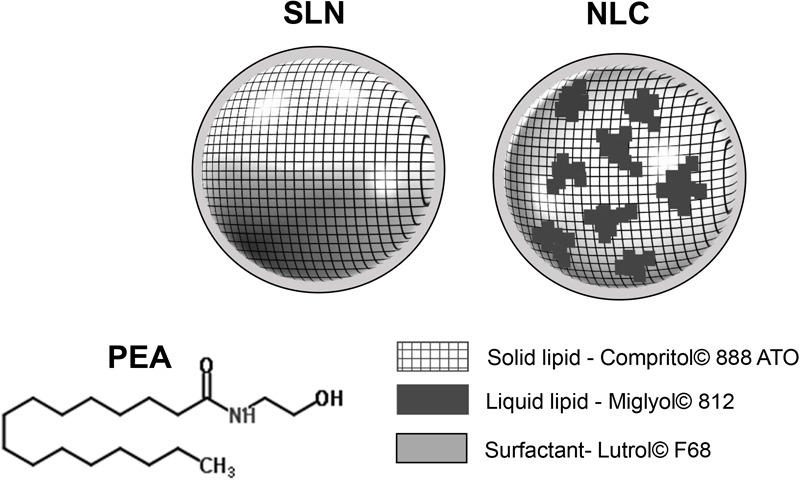
Structure of PEA (palmitoylethanolamide) and lipid nanoparticles (NPs). 1^st^ generation of lipid NPs (SLN, solid lipid nanoparticles) and 2^nd^ generation of lipid NPs (NLC, nanostructured lipid carriers).

Topical application of eye drops is the most common route of administration of ophthalmic drugs. Ophthalmic drug delivery, especially to the posterior eye segment, is one of the most challenging endeavors facing ocular pharmacologists. Ocular bioavailability of drugs which are administered by conventional solutions and suspensions is affected by a series of drug removal pre-corneal mechanisms which drastically reduce the amount of trans-corneal drug absorption (Puglia et al., 2015). Other unfavorable characteristics affecting the ocular delivery of drugs through the topical route are represented by the physiological barriers that limit significantly the drug absorption in the back of the eye (i.e., blood–aqueous barrier and the blood–retinal barrier) (Del Amo and Urtti, 2008; Bucolo et al., 2012). Therefore, frequent instillation of eye drops is necessary to obtain the expected therapeutic effect, even if discomfort and a decrease in patient compliance, especially in chronic therapy, are often observed (Battaglia et al., 2016). Delivery of drugs to the posterior eye is challenging, and there is an increasing need for therapeutic management of rapidly progressing retinal eye diseases, such as diabetic retinopathy, age-related macular degeneration, and optic neuropathy. Recently, an extraordinary effort has been made to develop new drug delivery systems, that prolong the corneal residence time to ameliorate the ocular bioavailability of ophthalmic drugs (Pignatello and Puglisi, 2011; Duan et al., 2015; Yu et al., 2015; Sanchez-Lopez et al., 2017). Nanostructured lipid carriers (NLC) are interesting nanosized particles belonging to the family of lipid nanoparticles (NPs), and NLC represent second generation NPs (**Figure 1**). They show important features advantageous for ocular application, such as controlled drug release, high drug loading, good bioavailability and excellent tolerability (Araújo et al., 2011; Puglia et al., 2015). Many scientific evidences demonstrate the NLC ability to improve the interaction with the ocular mucosa, prolonging the corneal residence time of the loaded drug, thus producing, on the one hand, an increase of the ocular bioavailability and, on the other hand, a reduction of the local and systemic side effects. Therefore, NLC are currently studied as delivery systems for the treatment of most important ocular disorders, from the common ocular inflammation or infection to important diseases affecting the posterior eye segment (Luo et al., 2011; Balguri et al., 2016; Tronino et al., 2016). Palmitoylethanolamide (PEA) is an endogenous congener of the endocannabinoid anandamide (AEA) with a potent anti-inflammatory activity, which can be exploited in different pathological conditions and in a variety of biological systems including retina (Matias et al., 2006; Petrosino et al., 2010). There are several reports highlighting that PEA can exert beneficial effects in several retinal diseases such as diabetic retinopathy, glaucoma, etc. A recent paper showed that PEA attenuated the degree of retinal inflammation while preserving the blood–retinal barrier in diabetic rats (Paterniti et al., 2015). Moreover, systemic PEA treatment was found to be effective in ameliorating visual field of glaucoma patients while decreasing intraocular pressure (IOP), which is the main risk factor of glaucoma (Gagliano et al., 2011; Costagliola et al., 2014). Furthermore, topical ocular PEA treatment was recently found to be effective in attenuate ocular surface inflammation in humans (Di Zazzo et al., 2017). The pharmacological properties of PEA include effects upon mast cells, CB_2_ -like cannabinoid receptors, ATP-sensitive K^+^-channels, TRP channels and NF-κB. However, the most robust evidence is related to the activity of PEA on the nuclear receptor peroxisome proliferator-activated receptor α (PPARα) (Lo Verme et al., 2005; LoVerme et al., 2006). Despite the potential pharmacological applications of PEA, its clinical application is strongly compromised by an unfavorable pharmacokinetic profile. PEA is a poorly water-soluble molecule (**Figure 1**), this physical chemical property limits the development of PEA eye drops, therefore, the aim of the present study was to set up a new ophthalmic formulation using innovative nanoparticles in order to enhance ocular bioavailability of PEA. Technological characterization of the novel formulation (PEA-NLC) was performed, and NLC morphology was assessed by electron microscopy. Finally, an *in vivo* study was carried out to assess formulation tolerability and pharmacokinetic profile of PEA after ocular administration in rabbit eye.

## Materials and Methods

### Materials

Compritol^®^ 888 ATO (COMP), a mixture of mono-, di-, and triglycerides of behenic acid, was obtained from Gattefossè (Milan, Italy); Miglyol^®^ 812 (MIG), a mixture of caprylic/capric triglycerides, was obtained from Eigenmann & Veronelli S.p.A. (Milan, Italy) and Lutrol^®^ F68 was provided by BASF Chem-Trade GmbH (Burgbernheim, Germany). Micronized PEA was a kind gift from Epitech Group (Milan, Italy). All the other chemicals and reagents were of the highest purity grade commercially available and were used as received.

### Lipid Nanoparticles Preparation

Palmitoylethanolamide-loaded NLC were prepared by high shear homogenization (HSH) method, following the procedure previously reported (Giannavola et al., 2003). Briefly, MIG (0.375 g) and PEA (0.260 g) were added to 1.5 g of molten COMP (80°C) and the mixture was stirred to obtain a dispersion. The molten lipid phase was dispersed in 25 mL of hot (80°C) saline containing Lutrol^®^ F68 (0.4% w/v), by using a high shear homogenizer (UltraTurrax T25; IKA-Werke GmbH & Company KG, Germany) at 13,500 rpm for 10 min. The hot dispersion was then cooled by dilution in 25 mL of additional water at 4°C. The resulting dispersion was then subjected to high-speed homogenization (8000 rpm) for 5 min. Unloaded NLC were prepared by the same procedure without adding PEA.

### Particle Size and Zeta Potential Measurement

NLC mean hydrodynamic diameter (mhd) and Zeta potential were determined using a Zetamaster instrument (Malvern Instrument, Ltd., Sparing Lane South, Worcs, United Kingdom), equipped with a solid state laser having a nominal power of 4.5 mW with a maximum output of 5 mW at 670 nm. All the measurements were carried out using a 90° scattering angle at 20 ± 0.2°C. Before measurements of particle size and Zeta potential, 10 μL of each sample suspension was diluted in 2 mL of ultrapure water, previously filtered through a 0.2 μm Acrodisc LC 13 PVDF filter (Pall-Gelman Laboratory, Ann Harbor, MI, United States).

### Morphological Studies

NLC morphology was evaluated by means of transmission electron microscopy (TEM) (Philips EM 400T microscope, Eindhoven, Netherlands) and field emission scanning electron microscopy (FE-SEM) (LEO 1525 equipped with a GEMINI column, ZEISS, Germany). TEM samples were prepared by deposition of a drop of diluted (100 folds) NLC suspension on the surface of a 200 mesh Formvar^®^-coated copper grid (TAAB Laboratories Equipment, Ltd., Aldermaston, England) and letting drop to evaporate at room temperature overnight. To investigate particle internal morphology by FE-SEM, the suspension was lyophilized, then particles were broken up in dry ice and, after CO_2_ complete vaporization, re-dispersed in water. Specimens were prepared by deposition of diluted (100 folds) NLC suspension onto an aluminum specimen stub covered with a double sided adhesive carbon disk. After water vaporization at room temperature, samples were sputter coated with chromium prior to imaging (Quorum Q150T ES East Grinstead, West Sussex, United Kingdom). Coating was done at 120 mA for 30 s.

### Ocular Pharmacokinetics

Experimental protocol was approved by the Institutional Animal Care and Use Committee of the University of Catania (Catania, Italy) and complied with the statements of Association for Research in Vision and Ophthalmology (ARVO) for use of animals in ophthalmic and visual research. New Zealand rabbits (weight 2.0–2.5 kg) were purchased from Envigo (San Piero al Natisone, Udine, Italy). Rabbits were housed in standard conditions, with free access to food and water, in a light-controlled room at controlled range of temperature (22 ± 1°C) and humidity (10–30%). Rabbits were sacrificed by intravenous administration of 0.3 ml/kg of Tanax^®^ (Intervet, Milan, Italy), after sedation with an intramuscular administration of 10 mg/kg of Zoletil^®^ (Virbac, Milan, Italy).

Rabbits were randomly assigned to two experimental groups (*n* = 8 per group), receiving single ocular topical administration (30 μL) of PEA-NLC and PEA suspension, respectively.

Palmitoylethanolamide concentration was 0.05% w/v, corresponding to [PEA] = 17.4 mM, both in the NLC formulation and suspension. A different set of animals (*n* = 6) was used to determine PEA content in tissues of control animals, that received topically 30 μL of unloaded NLC (*n* = 3) and pH 7.4 PBS (*n* = 3). Animals were sacrificed at 30, 60, 120, and 180 min after ocular topical administration of PEA-NLC and PEA suspension. Eyes were enucleated and ocular tissues (lens, humor vitreous, and retina) were then collected. Tissue samples were stored at -80°C till quantitative analysis of PEA. The following pharmacokinetics parameters were determined: peak eye tissue concentration (C_max_), time of peak of eye tissue concentration (T_max_), area under the curve (AUC_0-180_) of eye tissue building the curve (concentration of PEA [PEA] vs. time curve from 0 to 180 min). PEA concentration values in the tissues of treated animals were normalized to PEA content in tissue of control animals.

### Ocular Tolerability

The potential ocular irritancy and/or damaging effects of unloaded NLC and PEA-NLC formulations were evaluated according to a modified Draize’s test in a separate set of animals (4 animals/group) (Leonardi et al., 2014). Analysis was carried out using a slit lamp (mod. 4179 T Sbisà, Florence, Italy). Formulations (30 μL) were topically administered in the right eye every 30 min for 6 h (12 treatments). Congestion, swelling, and discharge of the conjunctiva were graded on a scale from 0 to 3 (0 = normal; 1, 2 and 3 = discrete, moderate and intense dilatation of conjunctival vessels, respectively), 0 to 4 (0 = normal; 1, 2, 3 and 4 = discrete, moderate, intense, intense + lid closure conjunctival swelling, respectively), and 0 to 3 (0 = normal; 1, 2 and 3 = discrete, moderate and intense discharge, respectively). Iris hyperemia was graded on a scale from 0 to 4 (0 = normal, 1 = discrete dilatation of iris vessels; 2 = moderate dilatation of iris vessels; 3 = intense iridal hyperemia with flare in the anterior chamber; 4 = intense iridal hyperemia with flare in the anterior chamber and presence of fibrinous exudates). Corneal opacity was graded on a scale from 0 to 4 (0 = normal, 1 = some spots of opacity; 2 = diffuse cortical opacity; 3 = cortical and nuclear opacity; 4 = intense opacity plus posterior subcapsular opacity). Formulations (30 μL) were topically administered in the right eye every 30 min for 6 h (12 treatments). At the end of the treatment, two observations at 10 min and 6 h were carried out in order to evaluate the ocular tissues. Observations were made by two independent observers in a masked way. Methylene blue staining was used in order to evaluate the corneal integrity, which allows an accurate determination of the extent of epithelial damage because of its poor diffusion through the stromal layer of the cornea.

### Ultra-High Performance Liquid Chromatography Tandem Mass Spectrometry (UHPLC-MS/MS)

Vitreous humor samples were filtered by 0.45 μM Phenex PTFE filters (Phenomenex, Castel Maggiore, BO, Italy), diluted 1:50 (v:v) with water (LCMS grade, Sigma Aldrich, Milan, Italy) and then directly analyzed by UHPLC-MS/MS. Lens and retina samples were brought to a temperature of -55°C and lyophilized overnight. Tissues were weighted, carefully crushed in an agate mortar and extracted in order to remove sample matrix interfering compounds. Extractions were performed by MetaboPrep-LC extraction kit (Theoreo, Montecorvino Pugliano, SA, Italy) according to the manufacturer’s instructions. Resulting solutions were analyzed as described below. The filtration and extraction methods were validated in control experiments and by spiking the tissues with a known amount of PEA before and after extraction. In all cases no interfering peaks deriving from the samples were detected. The recovery was in the range 97–115%. UHPLC-MS/MS analysis was carried out with a Shimadzu Nexera (Shimadzu, Milan, Italy) UHPLC consisting of two LC 30 AD pumps, a SIL 30AC auto-sampler, a CTO 20AC column oven, a CBM 20 A controller, and the system was coupled online to a triple quadrupole LCMS 8050 (Shimadzu, Kyoto, Japan) by a ESI source.

The separation was performed on a Ascentis Express^®^ HILIC column 150 × 2.1 μm, 2.7 mm (Supelco, Bellefonte, PA, United States) at a flow rate of 0.3 mL/min, employing as mobile phase (A) water 0.05 M HCOONH_4_ and (B) ACN, with the following gradient starting 0 min, 95% B, 0.01–3 min, 85% B, 3–6 min 85–40% B, isocratic for 30 s. Returning to 95% in 6 min. 2 microliters were injected. All additives and mobile phases were LCMS grade and purchased from Sigma Aldrich (Milan, Italy). The ESI was operated in positive ionization. MS/MS analysis was conducted in multiple reaction monitoring (MRM), employing as transitions: 300,4-62.2 (quantifier ion), 300,4-57.2 (qualifier ion). Q1 pre bias -16.0, -16.0 V, collision energy: -16.0, 30.0, Q3 pre bias -26.0 -24.0 V. Dwell time 25 ms. Interface temperature 300°C, Desolvation line temperature 250°C, Heat Block temperature 400°C, nebulizing gas, drying and heating gas: 3,10,10 L/min. All analyses were run in triplicate. Linearity of the method was assessed in the range of 0.01–250 ppb values were considered satisfactory (*R*^2^ = 0.9997). The low limit of detection (LOD) that was defined as the analyte concentration providing a signal-to-noise ratio of three, and it is considered the minimum concentration of analyte that could be confidently measured by the method (0.1 ppb).

### Statistical Analysis

GraphPad Prism (version 5.0; GraphPad Software, San Diego, CA, United States) was used for statistical analysis and graphical representation. All data are expressed as mean ± SD unless indicated otherwise. The results were analyzed using one-way ANOVA followed by Tukey–Kramer multiple comparisons test; Mann–Whiteny *U*-test was used for ordinal data. Data were considered statistically significant using as cut-off *p*-values < 0.05.

## Results

### Nanoparticles: Characterization and Electron Microscopy

**FIGURE 2:**
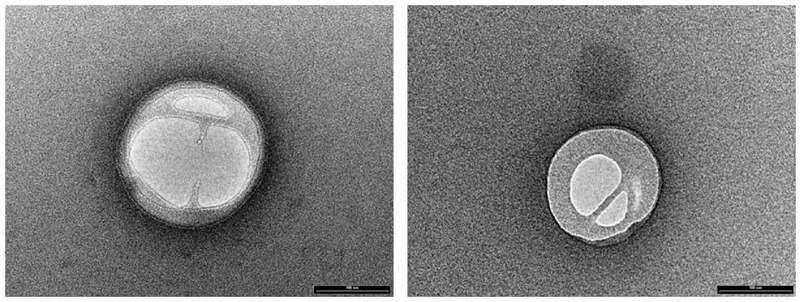
Transmission electron microscopy (TEM) photographs of PEA-NLC.

**FIGURE 3:**
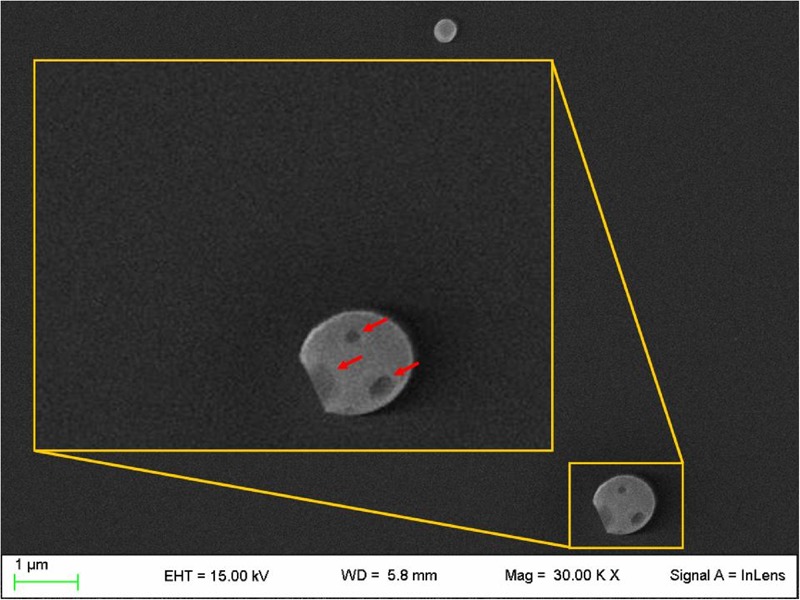
Field emission-scanning electron microscopy photographs of a fractured particle (PEA-NLC). Insert: enlargement of the fractured particle. Red arrows indicate the nano-compartments.

NLC loaded with PEA (PEA-NLC), prepared by HSH method, showed a mhd around 200 nm (208.6 ± 10.2 nm) with a polydispersity index (PDI) of 0.18. Unloaded NLC showed a lower mhd (171.3 ± 11.8) but a larger PDI (0.26). Both suspensions had a negative Zeta-potential higher than |20| mV, which should guarantee adequate suspension stability (Muller et al., 2002). Steric hindrance, due to presence of Poloxamer (Lutrol^®^ F68) on particle surface, might further contribute to the colloid stability. NLC suspensions had a pH of 7.64, compatible with ocular application, while osmolarity (242 mOsm/kg) had to be adjusted with the addition of 174 mg of NaCl to achieve isotonicity (300 mOsm/kg). Electron microscopy showed a particle size compatible with the one estimated by photon correlation spectroscopy data (**Figure 2**). The examination of particle internal structure at high magnification (140.000X) evidenced the presence of low-electrondense compartments within the matrix (**Figure 2**). These low-electrondense compartments had the tendency to merge together during sample heating by the microscope electron beam. **Figure 3** shows one fractured particle where it is possible to observe oil nano-compartments (red arrows).

### *In Vivo* Studies

**FIGURE 4:**
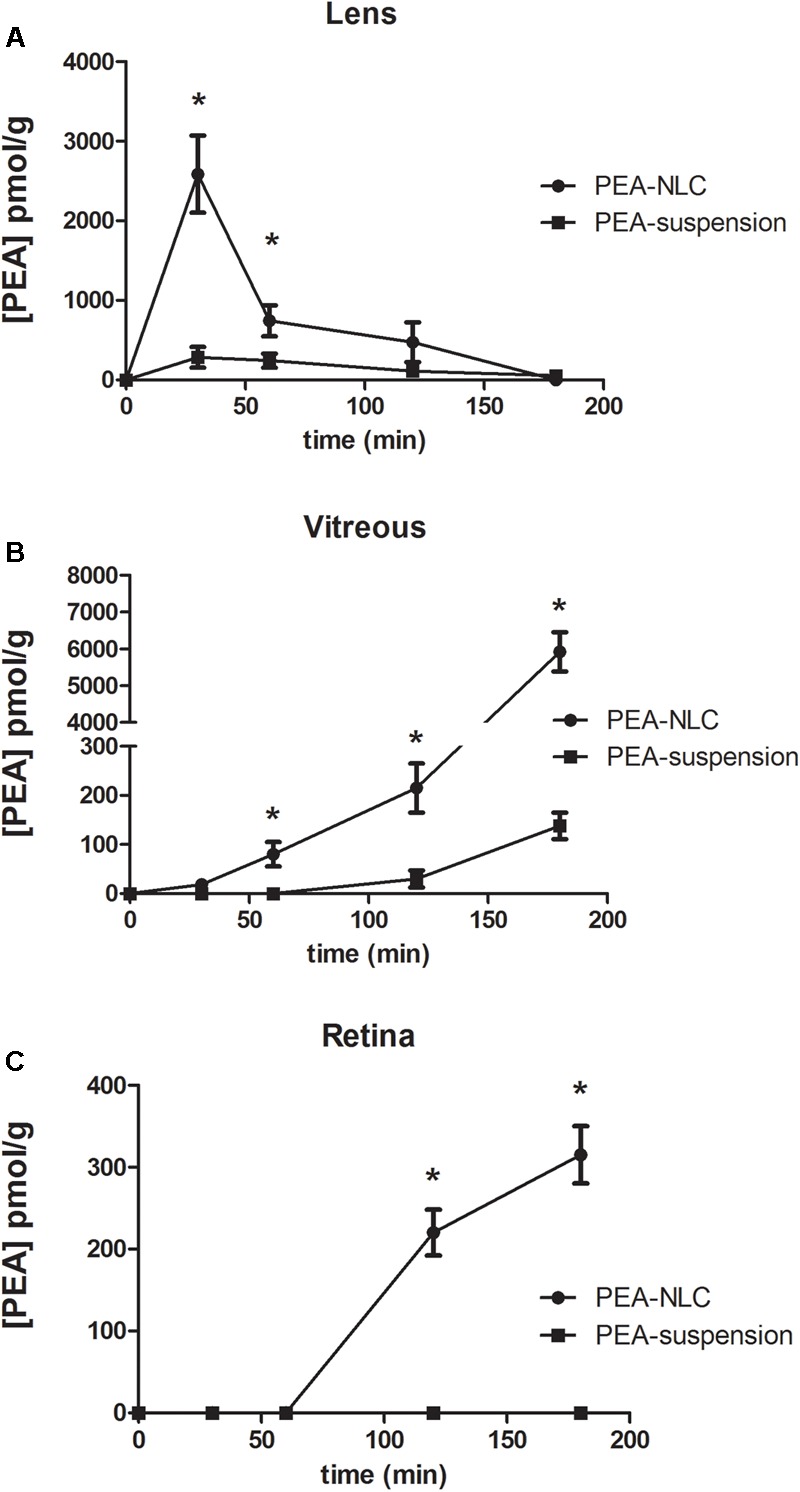
Levels of PEA vs. time curves in lens **(A)**, vitreous **(B)**, and retina **(C)** after treatment with PEA formulations. ^∗^*p* < 0.05. PEA-NLC vs. PEA-suspension.

The pharmacokinetic profile of a single 30 μL topical administration of 0.05% PEA encapsulated in lipid NPs (PEA-NLC) was compared to the same dose of PEA aqueous suspension (PEA suspension) [0.05% w/v in phosphate buffered saline (PBS); pH 7.4]. PEA had a C_max_ in the lens about ninefold higher when administered as lipid nanoparticles with respect to the suspension; and AUC_0-180_ of PEA-NLC was about fivefold greater (**Figure 4A** and **Table 1**) than AUC_0-180_ PEA-suspension. In fact, after absorption in the anterior segment of the eye, PEA, formulated as aqueous suspension, reached the vitreous, bearing a C_max_ = 138 ± 30 pmol/g, 180 min after the administration of the PEA suspension (**Figure 4B** and **Table 1**). Furthermore, the C_max_ of PEA in rabbit vitreous, 180 min after topical administration of PEA-NLC was about 43 fold higher (*p* < 0.01) than C_max_ reached after administration of PEA-suspension (**Figure 4B** and **Table 1**). Worthy of note, PEA, administered as aqueous suspension, did not reach detectable levels in the retina (**Figure 4C** and **Table 1**). On the contrary, PEA encapsulated in NPs (PEA-NLC) reached detectable levels in rabbit retina (C_max_ = 315 ± 70 pmol/g) (**Figure 4C** and **Table 1**). Ocular tolerability of blank NLC and PEA-NLC was assessed in rabbit eye. Both formulations, unloaded NLC and PEA-NLC, were well-tolerated and the score for each parameter was zero at all time of observations.

**Table 1:** Normalized pharmacokinetic parameters of topical ocular administration of PEA-NLC and PEA-suspension.

	Pharmacokinetics parameters						
	PEA-NLC			PEA-suspension			
	AUC (pmol⋅min/g)	t_max_ (min)	C_max_ (pmol/g)	AUC (pmol⋅min/g)	t_max_ (min)	C_max_ (pmol/g)	Basal [PEA] (pmol/g)
Lens	^∗^139639 ± 1532	30	2586 ± 485	27854 ± 2109	30	286 ± 135	409.6 ± 50.2
Vitreous	^∗^194673 ± 5957	180	5919 ± 541	5940 ± 567	180	138 ± 30	58.0 ± 4.7
Retina	22659 ± 786	180	315 ± 70	–	–	–	246.6 ± 28.8


^∗^*p* < 0.01 vs. PEA-suspension.

## Discussion

Pharmacokinetics data showed that a new PEA ophthalmic formulation (PEA-NLC) was able to deliver the molecule to the back of the eye. The mechanism by which PEA is locally delivered to the posterior segment from the pre-corneal area is likely to be attributed to several factors depending on NLC peculiar structure and morphology. The innovative NLC formulation was characterized by high stability, vitreo-retinal bioavailability and ocular tolerability. As previously reported (Tronino et al., 2016), the presence of low-electrondense spherical objects and their coalescence can be ascribed to the occurrence of MIG oily nano-compartments dispersed in the COMP solid matrix, as previously hypothesized (Muller et al., 2002). In fact, when nanoparticles are prepared with a mixture of solid and liquid lipids (e.g., oils), peculiar internal structures may be generated (Muller et al., 2002). These kinds of particles have been called NLC, and NLC can be differentiated as amorphous, imperfect, and multiple types, on the basis of internal structure features. The occurrence of one or another structure generally depends on the physicochemical properties and the relative amount of liquid excipient/s employed in the formulation. In the multiple type NLC, the relative high oil content and the reduced miscibility during particle solidification cause the separation of the oil, leading to formation nano-compartments within the solid matrix. The presence of these structures within the particle matrix has been visualized by TEM and confirmed by FE-SEM analysis (**Figures 2, 3**). NLC-formulation was further characterized by small particle dimension (208.6 ± 10.2 nm) with homogenous distribution of particle dimension (PDI = 0.18). Furthermore, both unloaded and loaded NLC showed a high stability to particle coalescence, bearing a negative Zeta-potential higher than |20| mV. Ocular pharmacokinetic of PEA loaded NLC (PEA-NLC) was compared to administration of aqueous suspension of PEA (PEA-suspension); the concentration of PEA was 0.05% in both ophthalmic formulations. Overall, our data shows that NLC significantly increased PEA ocular bioavailability in comparison to PEA delivered as aqueous suspension even in the posterior segment of the eye. Ocular tissues distribution of PEA suspension indicated that after instillation the drug followed a canonical direction from the anterior to the posterior segment of the eye; accounting mainly for a trans-corneal drug absorption mechanism (Bucolo et al., 2012) and, at least in part, to trans-scleral absorption, given PEA-suspension C_max_ = 138 ± 30 pmol/g in rabbit vitreous, 180′ after administration (**Figure 4B** and **Table 1**). PEA-NLC topical administration provided high retinal PEA levels (C_max_ 315 pmol/g), this amount is comparable to PEA levels (200 pmol) that were found to be pharmacological effective in two *in vitro* inflammation paradigms (Avagliano et al., 2016; Sarnelli et al., 2016). Additionally, we found that PEA basal levels (246.6 pmol/g) were superimposable to physiological PEA concentration found in healthy human retina (Chen et al., 2005).

In terms of safety, our results are in accordance with recent published studies on SLN and poloxamers from other groups. For instance, Gökçe et al. (2009) demonstrated that poloxamer 188, used to formulate SLN, was safe on the ocular tissues in a weight concentration ranging from 0.1 to 0.4%. Furthermore, safety of surfactant Lutrol^®^ F68, used in SLN, was proven by Araújo et al. (2011) that showed a full biocompatibility with the ocular tissues. Recently, it has been demonstrated that SLN stabilized by Poloxamer 188 are safe in rabbit eye (Leonardi et al., 2014).

In general, the mechanism by which drugs may be delivered to the posterior segment from the pre-corneal area is unknown. The evidence seems to indicate a non-corneal route, possibly involving conjunctival/scleral absorption followed by distribution to choroid, vitreous and retina, even though classical corneal absorption cannot be ruled out. Overall our data indicate that NLC could be able both to enhance PEA permeation through the conjunctival/scleral route and to facilitate the drug crossing through the corneal barrier. The latter mechanism is object of scientific debate since it seems that most NPs greater than 50 nm are readily immobilized by ocular mucus due to interactions with the mucin mesh (Lai et al., 2009; Schopf et al., 2015). Indeed, the ocular residence time of such trapped NPs should be related to the rapid turnover rate of the ocular mucus, typically in the order of seconds to minutes (Bala et al., 2004). We hypothesize that due to the characteristics of lipid shell, surrounded by the surfactant layer, our NLC by-pass tear mucin barrier and easily reach corneal glycocalyx layer. Therefore, penetrating the mucus layer, PEA-NLC guaranteed enhanced PEA delivery to ocular tissues, improving drug pharmacokinetics profile. In fact, it is well-known that mucin fibers are densely coated with negative charges and therefore they bind with high avidity positively charged particles by means of polyvalent adhesive interactions, including hydrophobic and electrostatic forces (Schopf et al., 2015). This feature represents a particularly challenging problem for positive charged NPs, designed to deliver drugs and genes (Ponchel et al., 1997).

Conversely, on the basis of previous reports (Bhat et al., 1996; Gökçe et al., 2009), free hydrophobic drug (PEA-suspension) diffusion would be dramatically affected by mucin layer, due to interaction with hydrophobic domain of mucus. Even though, after saturation of mucin binding sites, mucin fibers can act as merely inert fillers, as it was found in a model of mucus layer permeation (Bhat et al., 1995). Meaning that, after saturation of mucus layer binding sites, NLC and free drug would exhibit steady-state fluxes similar to those of buffer solution. Therefore, this effect of mucin barrier on steady-state fluxes of drug and particles explain why free PEA can cross the cornea, although to a less extent compared to PEA-NLC.

Despite the recent scientific literature reports many examples of interesting SLN (ancestors of NLC) application in ocular topical administration (Kakkar et al., 2015; Okur and Gökçe, 2017; Sanchez-Lopez et al., 2017), the results generated from these studies are very far to our data obtained with NLC (**Figure 1**). We demonstrated that the topical administration of PEA encapsulated in NLC resulted in a 40 and 100% increase of PEA levels in vitreous and retina, respectively, when compared with PEA suspension application.

## Conclusion

Taken together, our data indicate that the new ophthalmic formulation containing palmitoylethanolamide (PEA-NLC) has good ocular distribution, delivering high levels of drug in the back of the eye, after topical ocular administration. Therefore, this formulation may be useful in clinical practice to manage retinal diseases, indeed, clinical studies to evaluate this possibility may be warranted.

## Author Contributions

CP, CB, and RP made substantial contributions to the conception and design of the experiments and wrote the manuscript. CB, CBMP, GR, FG, FD, CP, RP, GP, PB, CO, ES, DS, BA, and PC participated in drafting and revising the manuscript. CP, RP, GP, PB, CO, ES, DS, BA, and PC made contributions on the technological field. CB, CBMP, GR, FG, and FD made contributions on the pharmacological field.

## Conflict of Interest Statement

The authors declare that the research was conducted in the absence of any commercial or financial relationships that could be construed as a potential conflict of interest.
